# Effect of Nano-Gefitinib on Solid Ehrlich Carcinoma via Targeting EGFR, RIPK2 Pathways, and Macrophage Reprogramming

**DOI:** 10.3390/ph18111747

**Published:** 2025-11-17

**Authors:** Neveen R. Ashoura, Hebatallah M. Saad, Enas I. El Zahaby, Alyaa R. Salama, Nihal E. Amer, Omnya Elhussieny, Hanan A. Edres, Hisham A. Nematalla, Salman A. A. Mohammed

**Affiliations:** 1 Department of Pharmacology, Faculty of Veterinary Medicine, Alexandria University, Alexandria 21944, Egypt; 2 Department of Pathology, Faculty of Veterinary Medicine, Matrouh University, Marsa Matrouh 51744, Egypt; heba.magdy@mau.edu.eg; 3 Department of Pharmaceutics, Faculty of Pharmacy, Delta University for Science and Technology, Gamasa 35712, Egypt; enas.elzahabi@deltauniv.edu.eg; 4 Department of Pathology, Faculty of Veterinary Medicine, Alexandria University, Alexandria 21944, Egypt; 5 Benha University Hospital, Benha University, Benha 13518, Egypt; 6 Department of Histology and Cytology, Faculty of Veterinary Medicine, Matrouh University, Marsa Matrouh 51744, Egypt; 7 Department of Biochemistry, Faculty of Veterinary Medicine, Alexandria University, Alexandria 21944, Egypt; 8 Department of Pharmacology and Toxicology, Faculty of Pharmacy, Damanhour University, Damanhour 22514, Egypt; hisham.nematalla@pharm.dmu.edu.eg; 9 Faculty of Pharmacy, The Research & Innovation Hub, Alamein International University, Alamein 51718, Egypt; 10 Department of Pharmacology and Toxicology, College of Pharmacy, Qassim University, Buraydah 51452, Saudi Arabia

**Keywords:** Ehrlich, gefitinib nanoparticles, macrophage reprogramming, oxidative stress, apoptosis, antioxidant activity, antineoplastic activity, cancer

## Abstract

**Background/Objectives**: Epidermal growth factor receptor-tyrosine kinase inhibitors (EGFR-TKIs) are a promising therapeutic avenue against mammary cancer. Thus, we investigated whether the EGFR inhibitor Nano-Gefitinib bilosome decreases Ehrlich tumor cells in a murine model, given that EGFR has been linked to carcinoma–macrophage crosstalk. **Methods**: Forty female mice were divided into control, Nano-Gefitinib, Ehrlich tumor and combination groups; the latter received Nano-Gefitinib treatment after tumor induction and lasted for 18 days. **Results**: Our results showed that Nano-Gefitinib ameliorated Ehrlich-induced hepatic injury, oxidative stress, and apoptosis in mice, as indicated by a significant reduction in serum level of hepatic enzymes, oxidative biomarkers (malondialdehyde and oxidized glutathione), total cholesterol, triglycerides, LDL, and BAX, along with an increase in antioxidant biomarkers, serum total protein, albumin, HDL, and hepatic antiapoptotic Bcl-2. A substantial reduction in tumor volume and size was noted in the combination group and was evidenced histopathologically by a reduction in tumor cell progression, mitotic activity, and giant cell formation. In addition, Nano-Gefitinib significantly inhibited EGFR/p-AKT/ERK1/2/RIPK2/NF-κB with subsequent suppression of TGF-triggered M2 macrophage reprogramming, evidenced by the lowered protein expression of the M2 surface markers CD163 and decreased M2 protein expression (Fizz1, MMPs, and VEGF). Additionally, Nano-Gefitinib significantly increased M1 macrophage phenotype, evidenced by the upregulation in the immunoexpression of the CD68, in addition to increasing CD8 and caspase-3 and decreasing CD4, with VEGF immunoreactivity in the combination group. **Conclusions**: Gefitinib biosomes encouraged macrophage polarization, apoptosis, and reduced inflammation, with a subsequent decrease in tumor volume.

## 1. Introduction

Mammary cancer is the most common type of cancer diagnosed worldwide and the leading cause of cancer-related mortality in both women and animals [1]. Breast cancer risk variables include gender, age, histology, genetics, reproductive risk factors, and personal history [2]. Ehrlich tumors are undifferentiated malignant neoplasia that has a high transplantable capacity and proliferative activity. Originally derived from a spontaneous mouse mammary adenocarcinoma, it can be propagated as both ascitic and solid tumor forms. They displayed rapid growth, robust angiogenesis, and reproducible host–tumor interactions, making it a versatile and reproducible experimental platform for evaluating nanomedicine-based anticancer compounds [3]. According to several studies, solid tumor models, including Ehrlich’s ascetic carcinoma (EAC), are useful tools for investigating biological processes in cancer and assessing the impact of possible cancer medications [4,5,6].

The cell surface-expressed epidermal growth factor receptor (EGFR) is a member of the tyrosine kinase-associated receptor family. EGFR is activated in healthy tissue by transforming growth factor-α (TGF-α) and epidermal growth factor (EGF) [7]. Because it inhibits apoptosis, promotes angiogenesis, inhibits cellular proliferation, and promotes metastasis, EGFR activation contributes to malignant transformation and tumor growth [8,9]. Individuals with metastatic carcinoma who have not responded to cytotoxic chemotherapy have shown a notable response to EGFR inhibitors. These findings propose that EGFR inhibitors may play a part in locally progressed metastatic cancer [10]. Numerous compounds, including cetuximab and Gefitinib, have been approved to disrupt EGFR tyrosine kinase signaling [11].

Gefitinib is a first-generation, orally active EGFR tyrosine kinase inhibitor (TKI) blocking downstream RAS/RAF/MEK/ERK and PI3K/AKT signaling responsible for proliferation and survival. Key limitations of free Gefitinib include low aqueous solubility, variable oral bioavailability, off-target systemic toxicities, and the development of primary and acquired resistance mechanisms. Such constraints have motivated preclinical efforts to improve its pharmacokinetic and tumor-targeting properties [12,13,14]. Nanotechnology-based formulations of small-molecule TKIs aim to increase solubility, protect the payload from premature metabolism, enable controlled or stimulus-responsive release, and exploit the enhanced permeability and retention effect for preferential tumor accumulation [15,16,17,18]. Gefitinib is a dibasic compound exhibiting pH-dependent solubility, with pKa values of 5.28 and 7.17. This property results in exceedingly low aqueous solubility across the physiological pH range. According to the Biopharmaceutical Classification System (BCS), Gefitinib is classified as a class II drug; enhancing its apparent solubility and dissolution kinetics is therefore a critical strategy to achieve a significant increase in systemic absorption and overall bioavailability [19,20]. Several studies were conducted to enhance solubility of poorly soluble drugs via a complex formation with cyclodextrin, spray drying, solid dispersion, and micelles entrapped technology [20,21]. Bilosomes are advanced lipid-based nanocarriers characterized by the strategic incorporation of bile salts into the vesicular bilayer, a modification that enhances their stability and permeation properties. The incorporation of bile salts as a bilayer component exerts a fluidizing effect on the membrane architecture. Their amphiphilic nature facilitates intercalation into the lipid bilayer, which disrupts lipid packing and reduces membrane micro-viscosity. This integration enhances bilayer hydration and increases molecular mobility, thereby diminishing membrane rigidity [22,23,24]. Collectively, these attributes establish bilosomes as a highly promising nanocarrier platform enhancing the bioavailability of poorly soluble drugs, motivating ongoing investigative efforts into their utility across a broadening range of therapeutic applications [23,25]. Muteeb et al. [26] showed that nanocarriers (e.g., liposomes, polymeric nanoparticles, lipid-based systems) can be functionalized with ligands that preferentially home to tissue-associated macrophages (TAMs) (e.g., via surface markers or receptors overexpressed in TAMs.)

The current investigation aimed to evaluate the potential effect of Nano-Gefitinib against Ehrlich-inoculated mice with an emphasis on its EGFR-mediated anti-angiogenic, antiproliferative, and apoptotic properties, as well as macrophage reprogramming. As far as we are aware, no information about the role of Nano-Gefitinib against EGFR-mediated macrophage reprogramming in the EAC tumor has been published in the literature.

## 2. Results

### 2.1. Bilosomes Characterization

#### Particle size, Zeta Potential, Poly Dispersity Index (PDI), and Drug Content

The average particle size of Gefitinib nanoparticle calculated by measuring the dynamic light scattering technique was 113.66 ± 2.23 nm, and the average zeta potential was −53.68 ± 1.36 mV, while the PDI was 0.31 ± 0.02. The average drug content was 3.22 ± 0.33 mg/mL (Table 1).

### 2.2. Transmission Electron Microscope (TEM) Results 

**Figure 1:**
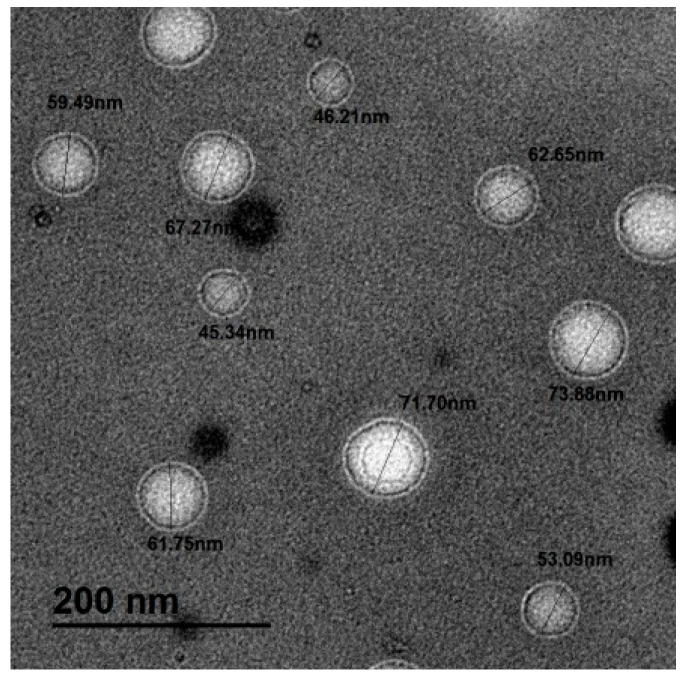
The transmission electron microscope (TEM) imaging of Gefitinib nanoparticles.

The TEM was capable of investigating the exact particle size and shape. The TEM examination of Gefitinib nanoparticles confirmed the homogenous monodispersing of bilosmes; spherical particles with well-defined dark walls, ranging from 45.34 to 73.88 nm (Figure 1).

### 2.3. FTIR Analysis Results

The FTIR of Gefitinib demonstrated a sharp characteristic peak at −3426 cm^−1^ corresponding to NH group; the peaks at −3045 and 2960 cm^−1^ are related to CH and CH2 stretching. The sharp peak at −1626 cm^−1^ corresponds to C=N, while the peak at −1578 cm^−1^ stands for C=C stretching [27,28].

The FTIR of NP Control illustrated a broad peak at −3439 cm^−1^ which can be related to OH stretching vibration of cholesterol, bile salt, and water; the broad peak indicates the extensive hydrogen bonding between the components of the formulation and, additionally, it is strong evidence for the successful incorporation of the bile salt into the lipid membrane [29].

The peak at −1642 cm^−1^ is related to the C=O stretching of bile salt. The presence of a peak at −1642 cm^−1^ is very characteristic of highly hydrated or H-bonded carbonyl groups. This is perfectly consistent with the bilosome structure, where the bile salt’s hydrophilic face and numerous OH groups interact with and hydrate the polar head group region of the lecithin bilayer. The presence of a peak at −2095 cm^−1^ is correlated to C≡C/C≡N stretching, which supports the extra-molecular interactions between bilosome components [30].

**Figure 2:**
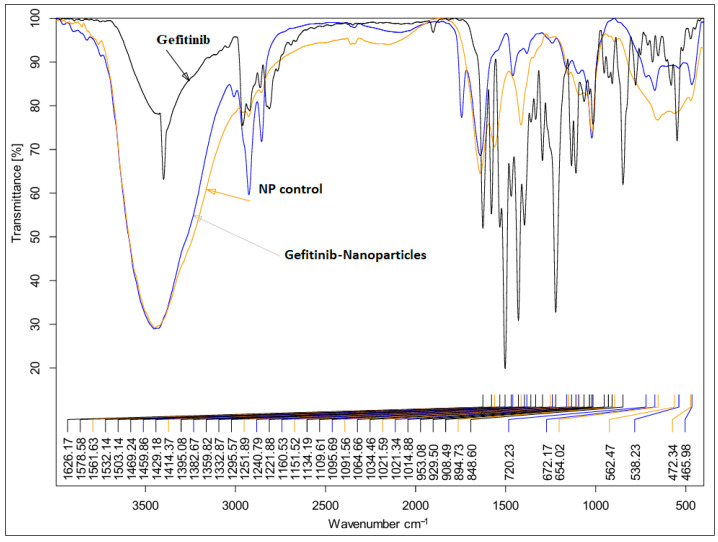
FTIR of Gefitinib, NP Control, and Gefitinib nanoparticles.

The FTIR of Gefitinib nanoparticles was quite similar to NP Control, with the absence of peaks in the fingerprint area of Gefitinib (below −1500 cm^−1^). Two peaks at −2958 and −2925 cm^−1^ were detected. The peak at −2099 cm^−1^ was observed, which was not present in the FTIR of Gefitinib. All these findings support the sequestration of Gefitinib successfully inside the bilosme (Figure 2).

### 2.4. Effect of Nano-Gefitinib on Tumor Volume and Tumor Weight in the Ehrlich Carcinoma Mice Model

**Figure 3:**
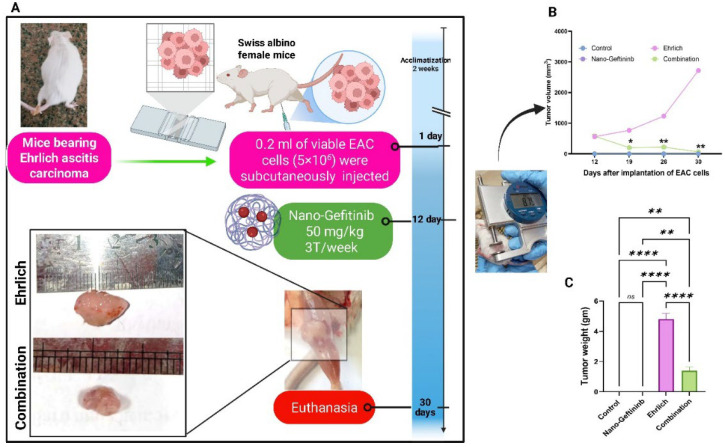
Effect of Nano-Gefitinib on Ehrlich carcinoma-mediated alterations on tumor volume and weight of mice. (**A**) Experimental design. (**B**) Tumor volume. (**C**) Tumor weight. Statistical significance represented by ns = non-significant, * *p* < 0.05, ** *p* < 0.01 and **** *p* < 0.0001.

As illustrated in Figure 3B,C, Ehrlich treated with the Nano-Gefitinib group displayed a significant decrease in tumor weight and volume in comparison with the Ehrlich group.

### 2.5. Effect of Nano-Gefitinib Against Ehrlich Carcinoma-Induced Alterations of Hepatic Function in Mice

**Figure 4:**
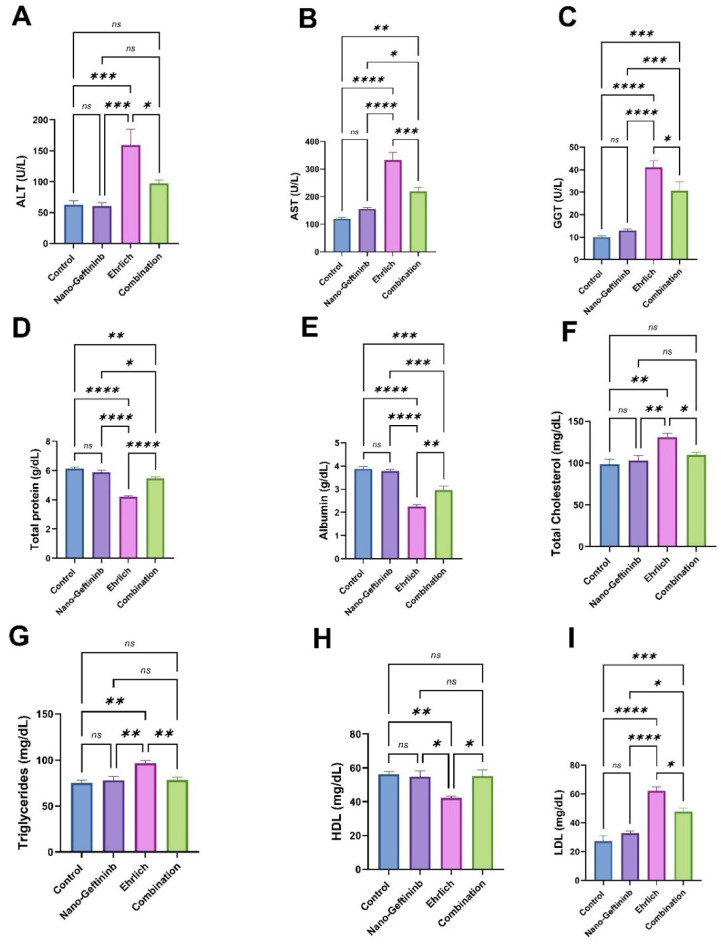
Effect of Nano-Gefitinib on Ehrlich carcinoma-mediated alterations in hepatic function biomarkers in mice serum. (**A**) Alanine aminotransferase (ALT, U/L). (**B**) Aspartate aminotransferase (AST, U/L). (**C**) Gamma glutamyl transferase (GGT, U/L). (**D**) Total protein (g/dL). (**E**) Albumin (g/dL). (**F**) Total cholesterol (mg/dL). (**G**) Triglyceride (mg/dL). (**H**) High-density lipoprotein (HDL, mg/dL). (**I**) Low-density lipoprotein (LDL, mg/dL). Statistical significance represented by ns = non-significant, * *p* < 0.05, ** *p* < 0.01, *** *p* < 0.001, and **** *p* < 0.0001.

As illustrated in Figure 4A–C, the hepatic enzymes (AST, ALT, and GGT) did not display any significant alterations in the Nano-Gefitinib-treated group compared to the control, even though there was a substantial upsurge in AST, ALT, and GGT levels in the Ehrlich group of 177%, 154.3%, and 310%, respectively, in comparison to the control group. Conversely, there was a substantial decrease in the levels of AST, ALT, and GGT (by 34%, 38.6%, and 25.3%, respectively) in the combination group compared to the Ehrlich group.

As presented in Figure 4D,E, the protein profile tests (total protein and albumin) did not display any obvious changes between the control and Nano-Gefitinib-treated group. On the other side, Ehrlich-bearing mice revealed a noteworthy decrease in the level of serum total protein and albumin (by 31% and 41.6%, respectively) as matched with the control mice. Contrariwise, the Ehrlich-treated-with-Nano-Gefitinib group displayed a major rise in total protein and albumin levels (by 28.5% and 30.8%, respectively) in contrast with the Ehrlich group.

As illustrated in Figure 4F–I, the lipid profile tests (total cholesterol, TG, HDL, and LDL) did not display any important alterations between the control and Nano-Gefitinib treated group. Meanwhile, the Ehrlich group verified a considerable increase in serum levels of total cholesterol, TG, and LDL by 33.13%, 28.5%, and 127.9%, respectively, in comparison to the control group. Conversely, the combination group revealed a significant decline in these biomarkers (by 16.1%, 18.8%, and 23.2%, respectively), compared to the Ehrlich group. Moreover, the HDL level showed a noteworthy decrease of 24.9% in the Ehrlich group when compared to the control group. In contrast, the combination group exhibited a significant elevation (30.8%) in the level of HDL relative to the Ehrlich group.

### 2.6. Effect of Nano-Gefitinib Against Ehrlich Carcinoma-Induced Hepatic Oxidative Stress in Mice

**Figure 5:**
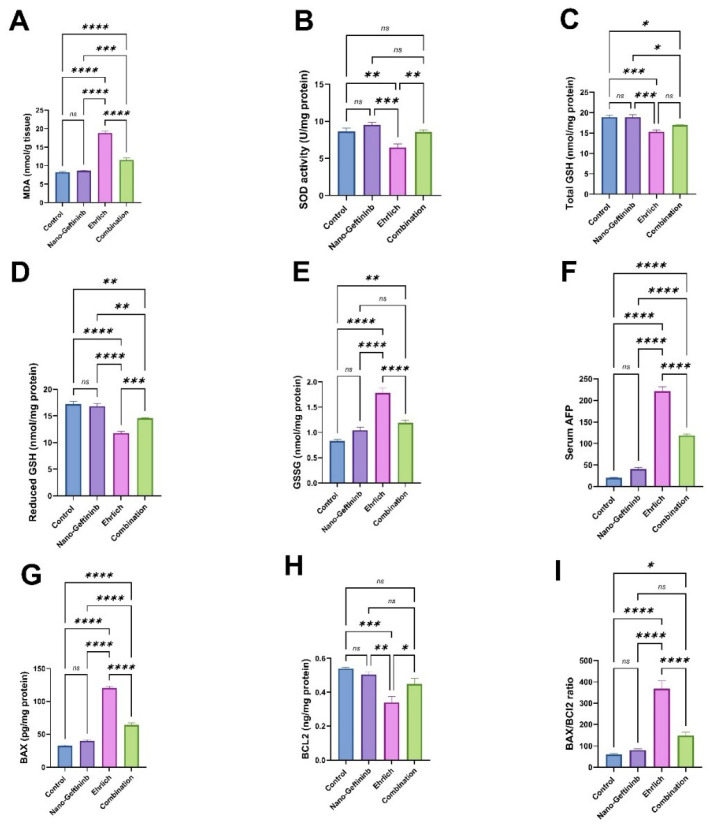
Effect of Nano-Gefitinib on Ehrlich carcinoma-mediated alteration in hepatic oxidative/antioxidant biomarkers and apoptotic markers in mice. (**A**) Malondialdehyde (MDA, nmol/g tissue). (**B**) Superoxide dismutase activity (SOD, U/mg protein). (**C**) Total glutathione (total GSH, nmol/mg protein). (**D**) Reduced glutathione (reduced GSH, nmol/mg protein). (**E**) Oxidized glutathione (GSSG, nmol/mg protein). (**F**) Serum alpha-fetoprotein (serum AFP). (**G**) BAX (BCL-2-associated X protein, ng/mg). (**H**) BCL2 (B-cell lymphoma 2, ng/mg). (**I**) BAX/BCL2 ratio. Statistical significance represented by ns = non-significant, * *p* < 0.05, ** *p* < 0.01, *** *p* < 0.001, and **** *p* < 0.0001.

As reported in Figure 5A–E, the hepatic oxidative and antioxidant biomarkers (MDA, SOD, total GSH, reduced GSH, and GSSG) did not exhibit any statistical change between the Nano-Gefitinib-treated mice and the control mice. Meanwhile, the Ehrlich-bearing mice displayed a significant upsurge in levels of MDA and GSSG (by 128.7% and 114.1%, respectively) with a substantial decline in levels of SOD, total GSH, and reduced GSH (by 25%, 18.8%, and 31.6%) compared to the control mice. Contrariwise, the Ehrlich-Nano-Gefitinib-treated mice exhibited a noteworthy decrease in MDA and GSSG levels (by 38.7% and 33.2%, respectively) with a significant upsurge in SOD, total GSH, and reduced GSH (by 31.9%, 24.2%, and 85.7%, respectively) matched to the Ehrlich group.

### 2.7. Effect of Nano-Gefitinib Against Ehrlich Carcinoma-Induced Alterations in Serum Tumor Marker in Mice

As mentioned in Figure 5F, the level of serum AFP did not show any important change between the Nano-Gefitinib and the control mice. Conversely, the Ehrlich group displayed a noteworthy increase in AFP level (994.4%) as matched to the control. In contrast, the level of AFP exhibited a significant reduction (46.2%) in the combination group compared to the Ehrlich group.

### 2.8. Effect of Nano-Gefitinib Against Ehrlich Carcinoma-Induced Hepatic Apoptosis in Mice

As presented in Figure 5G–I, there were insignificant changes in hepatic BAX, BCL2, and BAX/BCL2 ratio in mice administered with Nano-Gefitinib in relative to the control mice. However, the Ehrlich group revealed a notable increase in the level of BAX and BAX/BCL2 ratio (by 267.2% and 505%, respectively) with a noteworthy decline in the level of BCL2 (37.1%) as linked to the control group. On the other hand, the combination group presented a noteworthy decrease in BAX and BAX/BCL2 ratio (by 46.7% and 60.1%, respectively) with a noteworthy rise in the BCL2 (32%) level compared to the Ehrlich group.

#### 2.8.1. Effect of Nano-Gefitinib on Protein Expression of EGFR/p-AKT/ERK/RIPK2/NFκB in Ehrlich Mice Model

**Figure 6:**
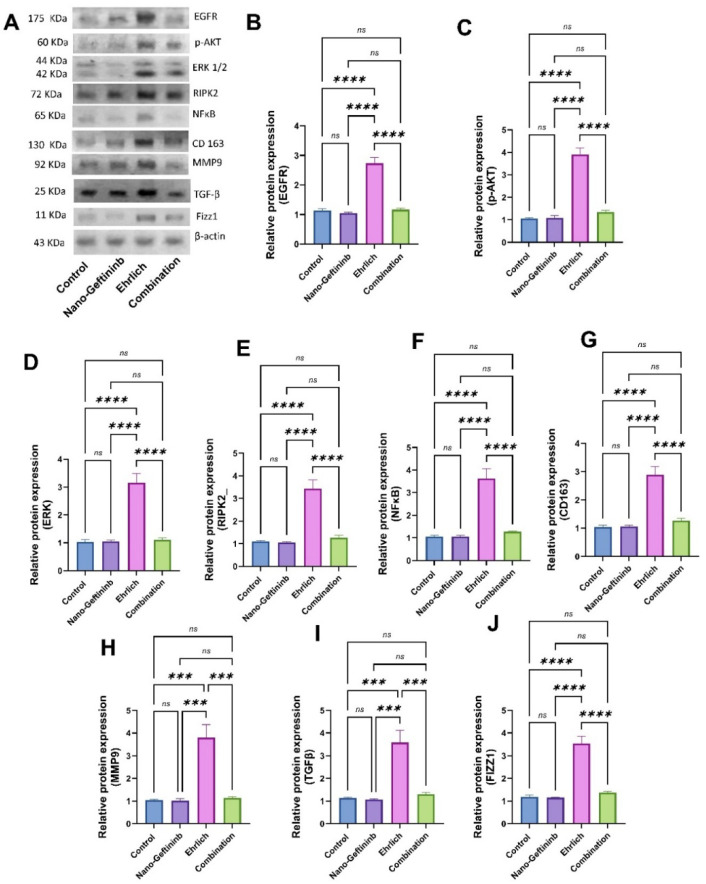
Effect of Nano-Gefitinib on Ehrlich carcinoma-mediated alteration on the protein expressions of the following markers in the tumor tissue homogenate of mice. (**A**) Bands of Western blot analysis. (**B**) Epidermal growth factor receptor (EGFR). (**C**) Phosphorylated AKT (p-AKT). (**D**) ERK1/2. (**E**) Receptor-interacting protein kinase 2 (RIPK2). (**F**) Nuclear factor of kappa (NF-κB). (**G**) CD163 macrophage marker. (**H**) Matrix metalloproteinase 9 (MMP9). (**I**) Transforming growth factor beta (TGF-β). (**J**) Found in inflammatory zone 1 (Fizz1). Statistical significance represented by ns = non-significant, *** *p* < 0.001, and **** *p* < 0.0001.

As shown in Figure 6A–F, both control and Nano-Gefitinib-treated groups did not display any significant differences in the protein expressions of EGFR, p-AKT, ERK, RIPK2, and NF-κB. Conversely, the Ehrlich group elicited a significant increment in the protein expressions of those parameters (by 461%, 272%, 205%, 211%, and 242%, respectively). However, the combination group exerted a noticeable decline in the protein expressions of EGFR, p-AKT, ERK, RIPK2, and NF-κB (by 57.5%, 65.7%, 64.7%, 62.9%, and 64.7%, respectively), compared to Ehrlich-inoculated mice.

#### 2.8.2. Effect of Nano-Gefitinib on Protein Expression of CD163/MMP9/TGF-β/Fizz1 in Ehrlich Mice Model

As illustrated in Figure 6A,G–J, both control and Nano-Gefitinib treated groups did not exhibit any significant alteration in the protein expressions of CD163, MMP9, TGF-β, and Fizz1. Conversely, the Ehrlich group exhibited a significant upregulation in the protein expressions of those parameters (by 176%, 260%, 216%, and 196%, respectively). However, the combination group exerted a noticeable decline in the protein expressions of CD163, MMP9, TGF-β, and Fizz1 (by 63.1%, 69.7%, 17.7%, and 61.4%, respectively), compared to Ehrlich-inoculated mice.

#### 2.8.3. Effect of Nano-Gefitinib on Histopathological Alterations in Ehrlich Mice Model

**Figure 7:**
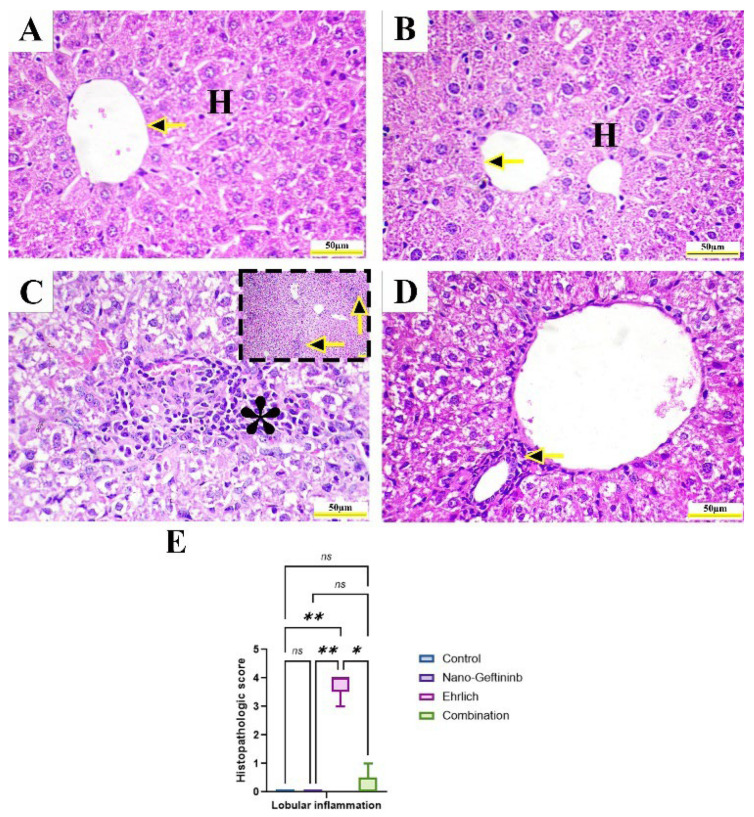
Effect of Nano-Gefitinib on histopathological changes induced by Ehrlich injection in hepatic tissue of mice. Hematoxylin and eosin (H&E). Bar (Squared **C**) = 100 µm. Bar (**A**–**C**) = 50 µm. (**A**) Control group and (**B**) Nano-Gefitinib group showing normally radiating hepatocytes (H) around the central vein (arrows). (**C**) Ehrlich group showing marked hepatic vacuolization and intense portal mononuclear inflammatory infiltrates (arrows and *). (**D**) Combination group showing mild portal inflammatory infiltrates. (**E**) Lobular inflammation histologic score statistical analysis. Statistical significance represented by ns = non-significant, * *p* < 0.05, ** *p* < 0.01.

Microscopic examination of hepatic tissue of both control and Nano-Gefitinib showed typical hepatic lobular architecture with normal central vein, portal area, and radiating hepatocytes (Figure 7A,B). On the other hand, the liver of Ehrlich-inoculated mice showed hepatic lobular inflammation with intense portal mononuclear inflammatory infiltrates and hepatocellular vacuolization (Figure 7C). Furthermore, the combination-treated mice revealed restoration of hepatic structure with mild portal mononuclear inflammatory infiltrates (Figure 7D). Statistical analysis of hepatic histopathologic score showed a marked increment in hepatic lobular inflammation in Ehrlich-inoculated mice (relative to control mice, this increment was downregulated after Nano-Gefitinib injection relative to the Ehrlich group by 94.7% (Figure 7E)).

**Figure 8:**
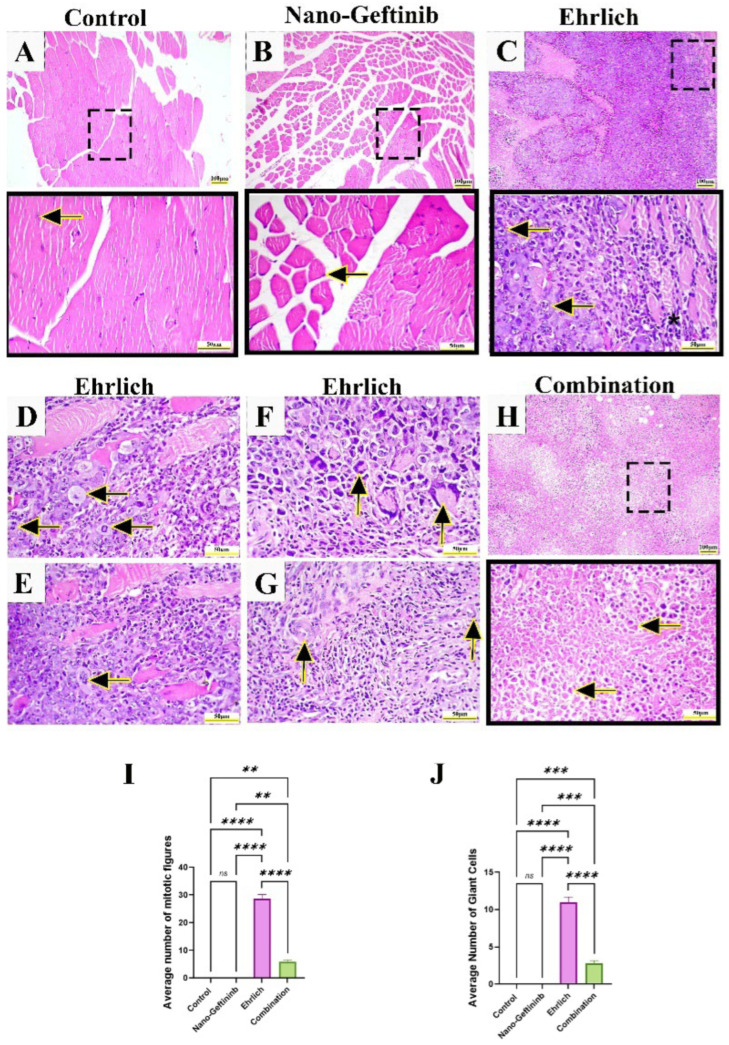
Effect of Nano-Gefitinib on histopathological changes induced by Ehrlich injection in thigh muscle of mice. Hematoxylin and eosin (H&E). Bar (**A**–**C**,**H**) = 100 µm and Bar (**D**–**G** and squared **A**–**C**,**H**) = 50 µm. (**A**) Control group and (**B**) Nano-Geftininb group showing typical muscle histoarchitecture with normal cross-striation and peripheral oval nuclei (arrow). (**C**) Ehrlich group showing solid sheets of malignant tumor cells invading muscle layers with minimal necrosis (Grade 1) and apoptosis (+) associated with intense inflammatory cells (*). (**D**,**E**) Atypical mitotic figures (arrows). (**F**) Multinucleated tumor giant cells (arrows). (**G**) Angiogenesis (arrows). (**H**) Combination group showing intense tumor necrosis (Grade 4) manifested by the appearance of ghost cells with a high apoptotic rate (+++) and dystrophic calcification. (**I**) Average number of mitotic figures. (**J**) Average number of tumor giant cells. Statistical significance represented by ns = non-significant, ** *p* < 0.01, *** *p* < 0.001 and **** *p* < 0.0001.

Muscle tissue of both control and control Nano-Gefitinib showed typical muscle histoarchitecture with normal cross-striation and peripheral oval nuclei (Figure 8A,B). Conversely, microscopic examination of the Ehrlich group exhibited massive muscular geographic necrosis infiltrated by solid sheets of tumor cells of high-grade malignancy characterized by polymorphic cells with bizarre-shaped nuclei and one or more prominent nucleoli (Figure 8C) with intense inflammatory infiltrates. Additionally, atypical mitotic figures (Figure 8D,E), scattered multinucleated tumor giant cells (Figure 8F), and angiogenesis (Figure 8G) were observed, indicating a high grade of cellular proliferation and rapid expansion of the tumor. Subsequently, histopathologic scoring of this group was significant angiogenesis (+++), limited necrosis (grade 1), and noticeable inflammatory infiltration (+++) with a high number of mitotic figures and giant cells that demonstrated the aggressive character of the tumor (Figure 8I,J and Table 2). The combination group showed ghost cells with dystrophic calcification, indicating excessive tumor necrosis (Figure 8H). Pleomorphism, angiogenesis, and mitotic figures were diminished with mild inflammatory infiltrates. This animal’s necrotic score was 4, which indicates widespread tumoral necrosis. The strong anticancer impact at this combined therapy was highlighted by the low (+) angiogenesis and inflammatory infiltration and the considerably elevated (+++) apoptosis (Table 2) with substantial decline in giant cell and mitotic figures relative to Ehrlich-treated mice (Figure 8I,J).

#### 2.8.4. Effect of Nano-Gefitinib on Immunohistochemical Expression of Caspase-3, Ki67, and VEGF1 in Ehrlich Mice Model

**Figure 9:**
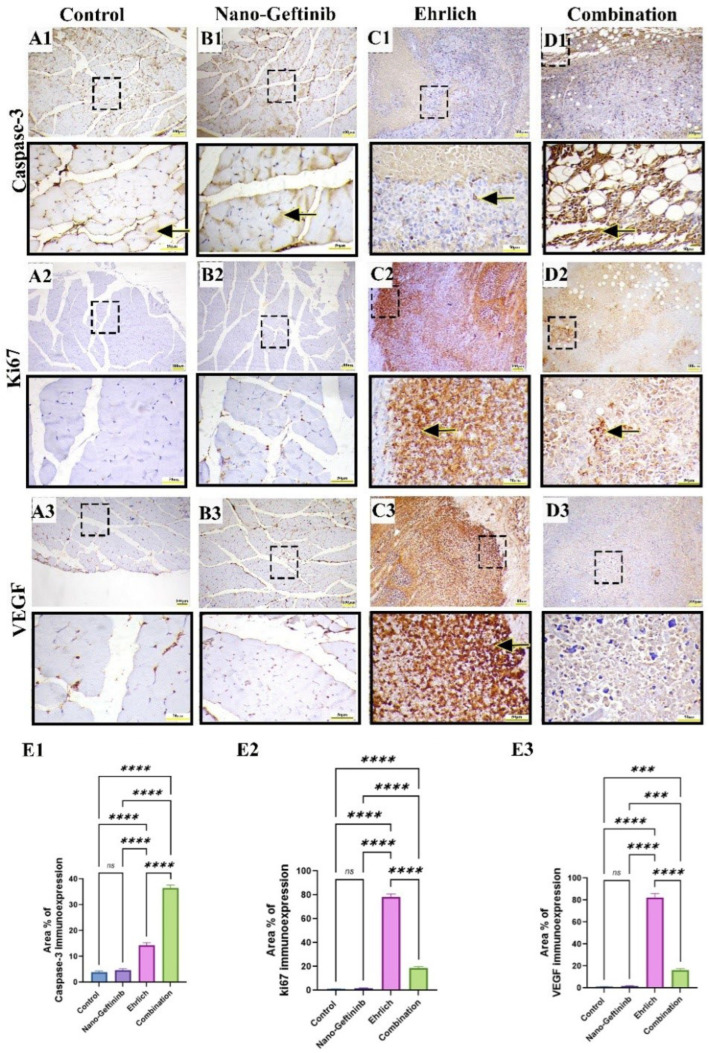
Effect of Nano-Gefitinib on apoptosis and tumor proliferation after Ehrlich inoculation in the thigh muscle of mice. Bar = 100 µm and Bar (squared boxes) = 50 µm. (**A1**–**D1**) Caspase-3 immunohistochemical expression of control (**A1**) and Nano-Gefitinib (**B1**) groups showing minimal expression (arrows), Ehrlich group (**C1**) showing mild brown expression (arrow), combination group (**D1**) showing intense positive immunoreactivity (arrow), (**E1**) Area% of caspase-3 expression. (**A2**–**D2**) Ki67 immunohistochemical expression of control (**A2**) and Nano-Gefitinib (**B2**) groups showing negative expression, Ehrlich group (**C2**) showing intense brown staining (arrow), combination group (**D2**) showing mild positive immunoreactivity (arrow), (**E2**) Area% of Ki67 expression. (**A3**–**D3**) Vascular endothelial growth factor (VEGF) immunohistochemical expression of control (**A3**) and Nano-Gefitinib (**B3**) groups showing negative expression, Ehrlich group (**C3**) showing intense brown staining (arrow), combination group (**D3**) showing minimal brown staining (arrow), (**E3**) Area% of VEGF expression. Statistical significance represented by ns = non-significant, *** *p* < 0.001, and **** *p* < 0.0001.

Microscopic examination of control and Nano-Gefitinib groups showed mild caspase-3 (Figure 9(A1–D1)) and negative Ki67 (Figure 9(A2–D2)) and VEGF (Figure 9(A3–D3)) immunoreactivity. On the other hand, the Ehrlich-inoculated group showed mild immunoreactivity for caspase-3 and marked immunoreactivity for Ki67 and VEGF1, indicating decreased tumor apoptotic death and increased tumor growth and vascularization. Meanwhile, the combination group exhibited intense immunoreactivity for caspase-3 and mild immunoreactivity for Ki67 and VEGF1, reflecting Gefitinib’s therapeutic activity in lowering tumor cell survival and neovascularization. Semiquantitative analysis of caspase-3, Ki67, and VEGF Area% immunoreactivity showed no significant difference between control and Nano-Gefitinib-treated mice. Conversely, a noticeable increase in caspase-3 and downregulation in Ki67 and VEGF Area% were recorded in the Ehrlich-Nano-Gefitinib group relative to the Ehrlich-inoculated group (Figure 9(E1–E3)).

### 2.9. Effect of Nano-Gefitinib on Immunohistochemical Expression of CD68 Macrophage, CD4 and CD8 Lymphocytes in Ehrlich Mice Model

**Figure 10:**
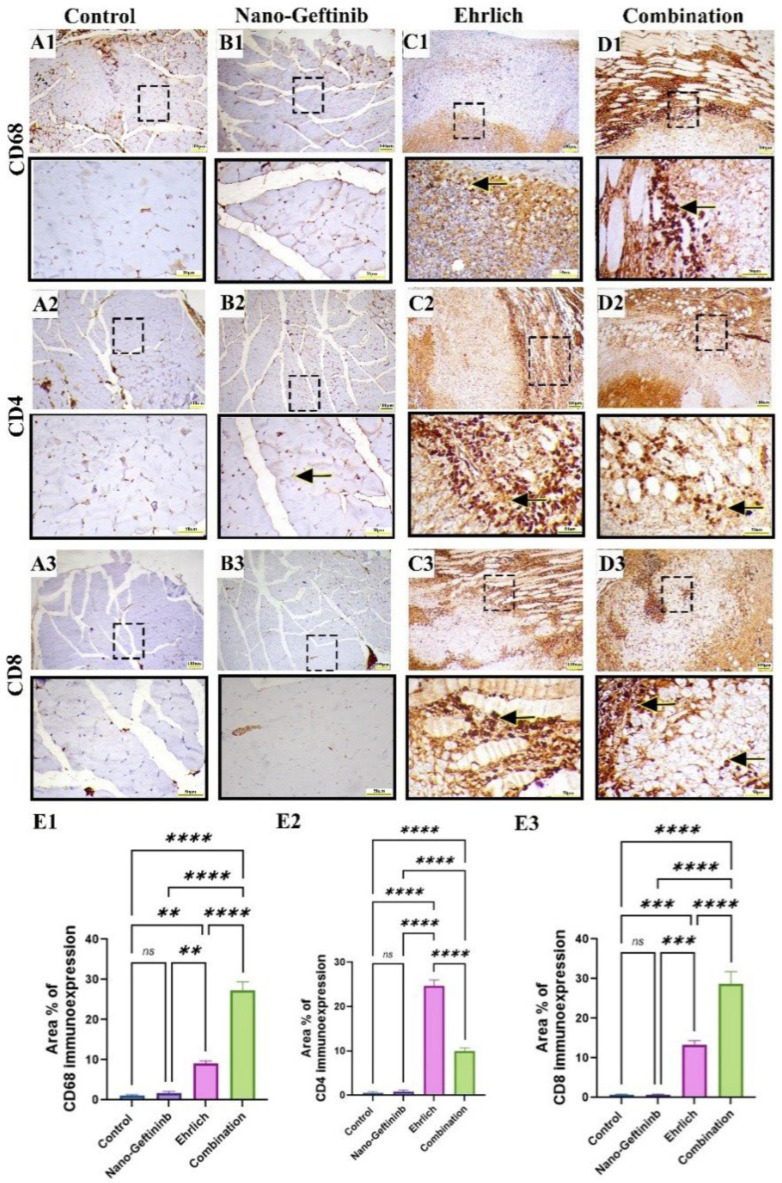
Effect of Nano-Gefitinib on apoptosis and tumor proliferation after Ehrlich inoculation in the thigh muscle of mice. Bar = 100 µm and Bar (squared boxes) = 50 µm. (**A1**–**D1**) CD68 immunohistochemical expression of control (**A1**) and Nano-Gefitinib (**B1**) groups showing negative expression, Ehrlich group (**C1**) showing mild brown expression (arrow), combination group (**D1**) showing intense positive immunoreactivity (arrow), (**E1**) Area% of CD68 expression. (**A2**–**D2**) CD4 immunohistochemical expression of control (**A2**) and Nano-Gefitinib (**B2**) groups showing negative expression (arrow), Ehrlich group (**C2**) showing moderate to intense brown staining (arrow), combination group (**D2**) showing mild positive immunoreactivity (arrow), (**E2**) Area% of CD4 expression. (**A3**–**D3**) CD8 immunohistochemical expression of control (**A3**) and Nano-Gefitinib (**B3**) groups showing negative expression, Ehrlich group (**C3**) showing mild brown staining (arrow), combination group (**D3**) showing moderate to intense brown staining (arrow), (**E3**) Area% of CD8 expression. Statistical significance represented by ns = non-significant, ** *p* < 0.01, *** *p* < 0.001, and **** *p* < 0.0001.

Microscopic examination of control and Nano-Gefitinib showed negative brown immunohistochemical staining for CD68 (Figure 10(A1,B1)). On the other hand, the Ehrlich-inoculated group showed mild immunoreactivity for CD68 (Figure 10(C1)), while the combination group exhibited intense CD68 immunoreactivity (Figure 10(D1)). Semiquantitative analysis of CD68 Area% immunoreactivity showed no significant alteration between control and Nano-Gefitinib-treated mice. Conversely, a marked increase in CD68 was noted in the combination group relative to the Ehrlich-inoculated group (Figure 10(E1)).

Microscopic examination of control and Nano-Gefitinib showed negative brown immunohistochemical staining for CD4 and CD8 lymphocytes (Figure 10(A2,A3,B2,B3)). On the other hand, the Ehrlich-inoculated group showed moderate to intense immunoreactivity for CD4 and mild immunoexpression of CD8 lymphocytes (Figure 10(C2,C3)), while the combination group exhibited mild immunoreactivity for CD4 and moderate to marked immunoexpression of CD8 lymphocytes (Figure 10(D2,D3)). Semiquantitative analysis of CD4 and CD8 Area% immunoreactivity showed an insignificant difference between control and Nano-Gefitinib-treated mice. Conversely, a marked decline in CD4 and a substantial increment in CD8 immunoreactivity were noticed in the combination group relative to the Ehrlich-inoculated group (Figure 10(E2,E3)).

### 2.10. Effect of Nano-Gefitinib on Ultrastructural Changes Induced by Ehrlich Inoculation in Mice Model

**Figure 11:**
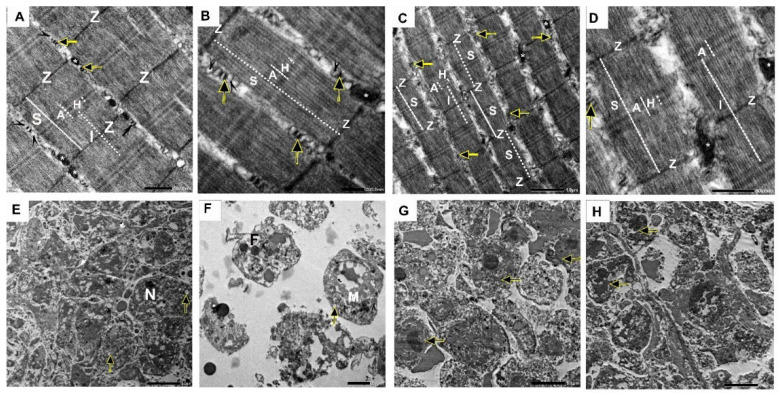
Effect of Nano-Gefitinib on ultrastructural alterations induced by Ehrlich carcinoma injection in mice under transmission electron microscope (TEM). (**A**) Longitudinal section of skeletal muscle of the control group showing the repetitive functional unit of the contractile apparatus of skeletal muscles, the sarcomere (S), extends from Z line (Z) to Z line. The skeletal muscle fibers have cross-striations of alternating more-electron-dense A bands (A, white line) and less-electron-dense I bands (I). The more-electron-dense A band is bisected by a narrow, less-electron-dense region called H zone (H, white dashed line). The I band is bisected by a dark transverse line, the Z line (Z). The sarcoplasm contains mitochondria (*). The T tubules (arrows) are associated with the sarcoplasmic reticulum cisternae (arrowheads), forming the triad of structures responsible for the cyclic release of calcium from the cisternae and its sequestration again, which occur during muscle contraction and relaxation (scale = 500 nm). (**B**) A higher magnification of the previous TEM showing the sarcomere (S), which extends from Z line to Z line, A band (A), H zone (H), mitochondria (*), T tubules (arrows), and cisternae of the sarcoplasmic reticulum (arrowheads) (scale = 200 nm). (**C**) Longitudinal section of skeletal muscle of Nano-Gefitinib group showing sarcomere (S), which extends from Z line to Z line (Z), the more-electron-dense A band (A) bisected by H zone (H), less-electron-dense I band (I), mitochondria (*), and the T tubule associated with the sarcoplasmic reticulum cisternae (arrows) (scale = 1 µm). (**D**) A higher magnification of the previous TEM showing the sarcomere (S), A band (A), H zone (H), I band (I), Z line (Z), mitochondria (*), and the T tubule associated with cisternae of sarcoplasmic reticulum (arrows) (scale, 500 nm). (**E**,**F**) Ehrlich group showing malignant tumor cells had a large nucleus (N) with condensed chromatin, ribosomes, mitochondria (M), lipid vacuoles (F), and numerous cytoplasmic vacuolization (arrows) (scale = 5 µm and 2 µm, respectively). (**G**,**H**) Combination group showing apoptotic and necrotic tumor cells (arrows) (scale = 5 µm).

The control negative group showed the normal ultrastructure of the skeletal muscles, which is composed of repetitive sarcomeres, the functional units of the contractile apparatus of skeletal muscles. The sarcomere extends from the Z line to Z line. The Z line is a dark transverse line that bisects the less electron-dense bands of the skeletal muscle fibers (I bands). The skeletal muscle fibers also contain areas of more electron-dense (A band), which is bisected by a narrow, less electron-dense region called the H zone. The alternating, more electron-dense A bands and less electron-dense I bands form the cross-striations of the skeletal muscle fibers. The sarcoplasm of the skeletal muscle fibers contains mitochondria for the energy needed for muscle contraction, and T tubules associated with the sarcoplasmic reticulum cisternae forming the triad of structures responsible for the cyclic release of calcium from the cisternae and its sequestration again, which occur during muscle contraction and relaxation (Figure 11A,B). The Nano-Gefitinib group is nearly similar to the control negative group; the skeletal muscle fibers are composed of sarcomeres, the alternating A and I bands are also present, in addition to the Z lines and H zones. The sarcoplasm contains mitochondria in addition to T tubules associated with sarcoplasmic reticulum cisternae (Figure 11C,D). Ehrlich’s group showed nearly rounded malignant tumor cells with a large nucleus (N), prominent nucleoli, and condensed chromatin. At the same time, the cytoplasm showed a deficiency of organelles with only free ribosomes, mitochondria (M), lipid vacuoles (F), and numerous cytoplasmic vacuolizations (Figure 11E,F). Furthermore, the combination group showed destruction of tumor cells characterized by cellular shrinkage and chromatin condensation, indicating apoptosis, while other cells showed membrane rupture, indicating necrosis (Figure 11G,H).

## 3. Discussion

Mammary cancer is the second most frequent illness worldwide, with over two million new cases reported each year [31,32]. Previous in vitro studies have demonstrated that Gefitinib effectively inhibits EGFR signaling and reduces tumor cell proliferation and macrophage-induced cytokine release [33,34,35]. In our in vivo Ehrlich solid carcinoma model, Nano-Gefitinib reproduced these effects, confirming a consistent inhibitory impact on the EGFR/p-AKT/ERK1/2/RIPK2/NF-κB axis and suppression of M2 macrophage polarization.

Bilosomes are nanoscopic vesicular carriers composed of a bilayer incorporating bile salts and amphoteric surfactants (lecithin). They are efficient platforms for drug delivery. Bile salts, which are endogenous surfactants, exhibit potent solubilizing and emulsifying properties. These characteristics are leveraged in pharmaceutical formulations to enhance the permeability of biological barriers and improve the aqueous solubility of hydrophobic drug compounds such as Gefitinib [30]. The present results indicated the success of bilosomes; the PDI was less than 0.5, indicating the monodispersion of particles. Additionally, high values of zeta potential (more than ±30 mV) support the electric repulsion between particles and, consequently, colloidal stability. The negative zeta potential is related to the presence of bile salt [36]. The slight variation between particle size estimated by dynamic light scattering and TEM examination can be explained as the dynamic light scattering technique measures the hydrodynamic diameter of the particles while TEM measures the exact particle size, which is the most accurate method for particle size estimation [37].

The TEM results confirmed the monodispersion and stable nanoparticles via the appearance of spherical particles with a well-defined wall (consisting of bile salt) and the particle size less than 100 nm with no evidence for particle agglomeration. The FTIR of Gefitinib nanoparticles were quite similar to NP Control with strong evidence for intermolecular hydrogen bond formations indicated by the appearance of a peak at −2099 cm^−1,^ which was not detected in the FTIR of pure drug. The evaluation of drug content indicated that almost all Gefitinib was entrapped successfully inside the bilosome structure

The tumor growth may affect the function of vital organs [38]. According to our results, ESC induced hepatic impairment, represented by a significant increase in serum liver enzymes, total cholesterol, TG, and LDL levels, with a significant reduction in serum total protein, albumin, and HDL levels in Ehrlich-bearing mice. These results were further confirmed by histopathologic investigation that revealed marked hepatocellular destruction and vacuolization with noticeable inflammatory cell infiltrates found in the Ehrlich group. Our results concur with those of Sakr et al. [39], Salem et al. [40], and Alamoudi et al. [41]. A recent study by Abd Eldaim et al. [42] and Elkholy et al. [43] stated that ESC induced hepatic injury, which was verified by a significant upsurge in the serum liver enzyme levels with a decrease in serum levels of albumin and total protein. Additionally, Abd Eldaim et al. [38] revealed the infiltration of Ehrlich tumor cells within internal organs. The possible explanation is the induction of oxidative stress in the livers of ESC-bearing mice [44,45]. Nevertheless, the treatment of Ehrlich mice with Nano-Gefitinib showed restoration of hepatic function parameters. These may be ascribed to the anti-inflammatory activity of Gefitinib by downregulating the NF-κB signaling pathway. Moreover, Li et al. [46] showed that Gefitinib significantly reduced oxidative damage and the expression of NF-κB, NOX enzymes, and MAPK (ERK, JNK, p38), thus enhancing cellular survival in normal lung cells.

Numerous studies have demonstrated that tumor growth can accelerate lipid peroxidation and create antioxidant disruptions in the tumor hosts’ critical organs [47,48,49,50]. Our data certified that ESC induced oxidative stress in hepatic tissues, which was reflected by a significant elevation in oxidative biomarkers (MDA and GSSG) with a significant decline in antioxidant biomarkers (SOD, total GSH, and reduced GSH). Our results are compatible with Ali et al. [51] and El-Shorbagy et al. [52] and [48,53]. However, the treatment of Ehrlich mice with Nano-Gefitinib showed significant enhancement against Ehrlich-induced hepatic oxidative stress. The antioxidant activity of Gefitinib was previously reported by K. P et al. [54], who displayed that Gefitinib showed hydroxyl radical scavenging and 2,2-diphenyl-1-picrylhydrazyl (DPPH) activity in vitro. Additionally, EGFR activity couples to NADPH oxidases, which generate ROS. Blocking EGFR by Gefitinib can lower NOX-driven ROS [55].

Alpha-fetoprotein (AFP) is a tumor marker commonly associated with malignant liver tumors, particularly hepatocellular carcinoma or benign liver diseases [56]. Our findings reported a noteworthy upsurge of serum AFP levels in the Ehrlich group. Our results were in line with those of Aldubayan, Elgharabawy, Ahmed, and Tousson [44]; Abd Eldaim, Tousson, El Sayed, Abd Elmaksoud, and Ahmed [42], and Hussein et al. [57], who explained that severe tumor development might induce oxidative damage that, consequently, increase the expression of AFP, which are incorporated in angiogenesis and support in the development of tumors. Moreover, our outcomes reported a noteworthy increase in BAX level with a significant decline in BCL2 in hepatic tissue in the Ehrlich group, indicating the apoptotic effect of ESC. Our results are in agreement with El-Shorbagy, Eissa, Sabet, and El-Ghor [52] and Hussein, El-khayat, and Farouk [57]. This might be attributed to ESC-induced cellular oxidative stress, causing P53 to activate cell-cycle arrest to provide time for self-mediated apoptosis [58]. Furthermore, our results showed that the Ehrlich-Nano-Gefitinib-treated mice exhibited amelioration in Ehrlich-induced hepatic apoptosis in mice. Our findings come in line with those of Wang et al. [59], who showed that Gefitinib diminished alveolar epithelial cell apoptosis and reduced fibrosis in bleomycin-induced pulmonary fibrosis in mice. The mechanism involves inhibition of EGFR signaling and reduction in oxidative stress, ultimately protecting normal lung tissue.

Microscopic examination of the Ehrlich group showed highly malignant tumor cells with high angiogenesis and mitotic activity, and little apoptotic activity, in addition to the formation of multinucleated tumor giant cells. Ultra-structurally, these tumor cells are characterized by a large nucleus, prominent nucleoli, and multiple cytoplasmic vacuoles with fat droplets. These findings are in line with previous studies [60,61]. Regarding the potential role of tumor giant cells in metastasis and chemoresistance [62], while histological results of the combination group showed the antitumor properties of Nano-Gefitinib, they were characterized by complete diminishment in tumor development and progression with a high apoptotic rate represented by shrunken cells with nuclear condensation.

A great proliferative percentage of people with ER-positive breast cancer is indicated by the breast cancer marker Ki-67 [63]. It is often used as an indicator for the growth of cancer cells in mice with Ehrlich [64]. The present study’s Ehrlich group showed minimal apoptotic death and strong proliferative activity, as shown by a substantial reduction in caspase-3 protein immunopositivity, alongside an observable increase in Ki-67 immunopositivity. These observations are parallel to earlier research [60]. The study’s conclusions of Nano-Gefitinib’s antitumoral effectiveness are linked to a substantial decline in the expression levels of Ki-67 with an increment in caspase-3 immunoreactivity.

Our results indicated a significant upregulation of EGFR, p-AKT, ERK2, RIPK2, NF-κB, CD163, Fizz1, MMP9, and TGF-β, accompanied by a downregulation of CD68 in the Ehrlich carcinoma model. The observed increase in EGFR and its downstream effectors p-AKT and ERK2 is consistent with the recognized role of EGFR signaling in promoting tumor growth, proliferation, and survival through activation of the PI3K/AKT and MAPK/ERK pathways [65]. The upregulation of RIPK2 and NF-κB further supports the involvement of innate immune signaling and inflammatory cascades in sustaining tumor progression and immune evasion [66]. In parallel, increased expression of the M2 macrophage markers CD163 and Fizz1 suggests a shift toward an immunosuppressive microenvironment that favors tumor growth [67]. The concomitant elevation of MMP9 and TGF-β aligns with their known roles in extracellular matrix degradation, angiogenesis, and metastasis, as well as immunosuppressive signaling within the tumor microenvironment [68]. Conversely, the downregulation of CD68, a pan-macrophage marker, may reflect a reduced proportion of classically activated macrophages relative to M2-polarized subsets, thereby reinforcing the dominance of an immunosuppressive tumor-associated macrophage (TAM) phenotype. Parallel to our result, a study by Mansour and Ibrahim [65] showed that Ehrlich inoculation in a murine model resulted in a remarkable increment in the protein expression of PI3K, p-Akt, and NFκB, while Kalish et al. [69] showed that Ehrlich injection suppressed the macrophage nitric oxide release in vivo and reprogrammed macrophages towards the M2 phenotype (CD206). In preclinical models, EGFR-targeted tyrosine kinase inhibitors quickly suppress PI3K-AKT, MAPK/ERK signaling in tumor cells, preventing tumor cell lines from proliferating in Swiss albino mice [70]. Maloney et al. [71] showed that Gefitinib reprogrammed macrophages by inhibiting RIPK2, which prevents invasion and metastatic extravasation induced by macrophages in osteosarcoma. Thus, in our study, Nano-Gefitinib markedly suppressed TGF-induced M2 macrophage reprogramming by inhibiting EGFR/p-AKT/ERK1/2/RIPK2/NF-κB, as demonstrated by decreased M2 protein expression (Fizz1, MMPs, and VEGF) and the protein expression of the M2 surface markers CD163. Furthermore, the enhancement of the M1 surface markers CD68’s immunoexpression demonstrated that Nano-Gefitinib markedly improved the M1 macrophage phenotype.

The stimulation of proangiogenic proteins, such as MMP-9 and VEGF, initiates the energy-dependent process of angiogenesis. The present investigation indicates that Ehrlich inoculation resulted in an increase in MMP-9 protein level and VEGF immunoreactivity, which were downregulated by Gefitinib. This study is parallel to a study by Mahmoud et al. [72] that reported a remarkable increase in VEGF and p-AKT in Ehrlich mice. Furthermore, research has shown that Gefitinib inhibits VEGF and MMP-9 [73], which stops breast cancer cells from angiogenesis and neovascularization.

According to our results, Ehrlich cells’ inoculation resulted in a substantial rise in CD4^+^ expression, more than CD8^+^ positive cells immunoreactivity. This is because the functions of CD4^+^ and CD8^+^ T lymphocytes in the development of breast cancer are opposed. Although they do so in distinct ways, CD4^+^ T cells may contribute to metastasis [74]. Conversely, CD8^+^ T cells are the predominant effector cell type and positively impact both patient clinical outcomes and antitumor immunity [75]. Additionally, macrophages affect how T cells polarize; M2 macrophages encourage the development of regulatory T cells [76]. In the present investigation, Nano-Gefitinib effectively increased CD8^+^ immunoreactivity more than CD4^+^ immunoreactivity in the Ehrlich-treated group. Diao et al. [77] reported that because Gefitinib-loaded PEG5k-Fmoc-NLG919 creates an immune-active milieu with more functional CD8^+^ T cells and less regulatory T cell infiltration, it may be able to suppress the formation of lung tumors more effectively in vivo than Gefitinib alone. The current study presents the first evidence in the Ehrlich carcinoma model that nano-formulated Gefitinib preferentially enhances macrophage reprogramming, with elevated CD8^+^ over CD4^+^ response. While no prior data exist in the Ehrlich tumor, similar nanoparticle immunotherapeutic strategies have not been used in other tumor models.

Future investigations should evaluate the long-term safety profile and nanotoxicity of Nano-Gefitinib after prolonged administration, as subtle toxic effects may require extended exposure periods to manifest. Moreover, dose optimization and pharmacokinetic studies are essential to correlate nanoparticle accumulation with therapeutic outcomes.

Mechanistically, it would be valuable to explore upstream and downstream regulators of RIPK2 and NF-κB, including TLR–MyD88–TRAF6 signaling and the potential involvement of autophagy-related proteins (p62, LC3, mTOR) in macrophage polarization. Advanced in vitro co-culture models using tumor cells and macrophages, alongside flow cytometry-based immune profiling, could further delineate how Nano-Gefitinib shifts the tumor microenvironment balance toward the M1 phenotype.

Finally, future studies could compare the efficacy of Nano-Gefitinib with other targeted therapies or checkpoint inhibitors (e.g., anti-FGL1/LAG-3 blockade) to determine whether combinatorial regimens yield synergistic benefits in cancer therapy.

## 4. Materials and Methods

### 4.1. Drugs and Chemicals

Gefitinib was obtained from (©Tokyo Chemical, Industry Co., Ltd., Tokyo, Japan). All chemicals were of good quality and analytical grade. Bile salt (ANXES, Barcelona, Spain), lecithin (Lipoid Kosmetik, Deutschland, Germany), cholesterol 95% (Alfa Aesar, Karlsruhe, Germany), chloroform (Alpha Chemika, Mumbai, India), and deionized water.

### 4.2. Preparation of Bilosomes

NPs control was prepared by a slight modification of the previously published thin film hydration method. Briefly, lecithin (2.4 g), bile salt (0.4 g), and cholesterol (0.6 g) were dissolved in a chloroform–ethanol mixture (10:1); the mixture was poured into a round-bottom flask and evaporated utilizing a rotary evaporator (60 °C/280 rpm) at low pressure (Heidolph, Schwabach, Germany). Then, pre-heated purified water (120 mL, 60 °C) was employed to hydrate the thin lipid layer with continuous agitation. The colloidal dispersion was further homogenized for 5 min at a speed of 10,000 rpm (IKA ULTRA-TURRAX^®^ T25, Staufen, Germany) and treated with ultrasound probe sonicator (Sonic, Vibra Cell, Newtown, CT, USA) for 5 min (10 s on\10 s off) 60% amplitude (130 W). The procedures were repeated with the addition of Geftinib (0.4 g) to the lipid mixture to formulate Gefitinib-Nano [24,78].

### 4.3. Bilosomes Characterization

#### 4.3.1. Particle Size, Zeta Potential, Poly Disperisty Index (PDI)

The hydrodynamic particle size, expressed as the mean diameter (MD), polydispersity index (PDI), and zeta potential, were determined for the Gefitinib-Nanoparticle formulations using a Zetasizer Nano ZS (Malvern Instruments, Malvern, UK) based on the principles of dynamic light scattering. All samples were diluted with deionized water to achieve a scattering intensity of ~300 kHz. The samples were thermally equilibrated at 25 °C for 5 min before measurement. Data are reported as the mean of three consecutive measurements per sample [79].

#### 4.3.2. Drug Content and % Entrapment Efficiency (EE)

The drug content of the colloidal dispersion was estimated by a spectrophotometric method [80]. A UV-visible spectrophotometer (Shimadzu UV-VIS spectrophotometer, UV-1900I, Kyoto, Japan) was employed for scanning the Gefitinib spectrum in the range of 200 to 400 nm with the aid of 3 mL quartz cuvette (light’s travel length 1 cm). Gefitinib stock solution was prepared in absolute alcohol; serial dilution was performed for the stock Gefitinib solution in distilled water (in the range of 0.4 to 20 µg/mL). The colloidal dispersion was properly diluted with distilled water and measured at 254 nm in triplicate.

The EE of bilosome was estimated according to the following equation:

$$EE\%=\left(\frac{Amount\;of\;drug\;entrapped}{amount\;of\;drug\;used}\right)\times 100$$

#### 4.3.3. Transmission Electron Microscope (TEM)

The primary particle size and nanoscale morphology of the Gefitinib nanoparticle were evaluated by transmission electron microscopy (TEM, JEM-2100F, JEOL Ltd., Akishima, Japan). Samples were prepared by depositing a dilute aqueous dispersion of the nanoparticles onto a grid. To enhance contrast for imaging, the samples were negatively stained with uranyl acetate (2% aqueous solution) for 3 min, blotted, and subsequently air-dried prior to insertion into the microscope column.

#### 4.3.4. FTIR Analysis

The functional groups were found utilizing an air-cooled DTG (deuterated triglycine sulfate) detector and infrared spectroscopy (FTIR; Perkin Elmer Fourier transform spectrometer, BRUKER, Portland, OR, USA). In a concise manner, Gefitinib, NPs Control, and Gefitinib nanoparticles. KBr was combined with each sample and compressed using a hydraulic press. We observed the disk at wavelengths between −4000 and −400 cm^−1^.

### 4.4. Experimental Animals

Mature female mice (Swiss albino) with average weights of 22–25 g were supplied by the National Institute of Ophthalmology in Giza, Egypt, and acclimatized for 2 weeks. They had been fed ordinary pellet chow produced by the EL-Nasr Chemical Company in Cairo, Egypt. There were no limitations on their access to water. The animals were given a week to adjust to the identical settings before the trial. The Institutional Animal Care and Use Committee (IACUC), Alexandria University, Egypt, authorized the experimental procedures under approval number (ALEXU-IACUC, 2025-3-10/338). All methods adhered to the ARRIVE standards [81].

### 4.5. Tumor Induction and Experimental Design

The cancer cell line was purchased from the National Cancer Institute (NCI), Pharmacology and Experimental Oncology Unit at Cairo University, Egypt. Tumoral cells were identified in the ascitic fluid during the first ten days. Disposable needles were used for entering the abdominal cavity and extracting the malignant cells, then counted by a hemocytometer [82]. The trypan blue dye exclusion method demonstrated that the cells exhibited over 99% vitality [83]. Subsequently, 0.2 mL of viable EAC cells (5 × 10^6^) was subcutaneously administered into the left thigh of the lower limb [84]. On the 12th day post-injection of the malignant cells, an 18-day therapeutic regimen of Nano-Gefitinib was applied.

Forty female adult mice were allocated into four groups (10 mice/group): (1) control group: mice were injected with 0.5 mL saline intraperitoneally. (2) Nanoparticle control group: Nano-Gefitinib was injected (50 mg/kg) intraperitoneally, three times per week [85]. (3) Ehrlich group: Mice were injected with 0.2 mL of live EAC cells subcutaneously. (4) Combination group: Twelve days post-Ehrlich inoculation, ESC mice received an intraperitoneal injection of Nano-Gefitinib at a dose level of 50 mg/kg, three times per week for 18 days [85] (Figure 3A).

### 4.6. Evaluation of Tumor Volume and Weight

Tumor volume was assessed 12 days post-inoculation (day 0) and every week throughout the 30-day study duration utilizing a digital caliper (Dasqua, Cornegliano, Italy). By using the formula below, the tumor volume (mm^3^) was assessed: in equation $V\;\left(Volume\right)=0.52\times {AB}^{2}$, *A* is the minor axis and *B* is the major axis [86,87]. Following the end of the experimental times, the mice were euthanized with isoflurane. After dissection, cleansing, and weighing using a digital balance, the tumor tissue was divided into two slices. The initial portion was cryogenically frozen in liquid nitrogen for protein analysis and thereafter stored at −80 °C. The second portion was fixed for histological and immunohistochemical examination in 10% buffered formalin.

### 4.7. Blood and Tissue Sampling

At the end of the trial using isoflurane euthanasia, blood samples were collected from the aorta in plain tubes and then left for coagulation for 30 min at the slop position. Blood tubes were placed in a centrifuge for 15 min, and then the serum was transferred to clean Eppendorf tubes for further biochemical studies. The liver of all mice were carefully dissected and then divided into two fragments; part of the tissue was fixed in 10% neutral buffered formalin for histopathological and immunohistochemical analysis. The other part was washed in saline for homogenization and kept at −80 °C for further biochemical analyses.

### 4.8. Evaluation of Hepatic Performance Using Serum Biochemical Markers

The levels of hepatic enzymes, alanine aminotransferase (ALT), and aspartate aminotransferase (AST) were calorimetrically measured [88] and gamma glutamyl transferase (GGT) was measured according to Szasz [89] (kits from Biolabo, Maizy, France). The levels of serum total proteins, albumin, total bilirubin, total cholesterol (TC), triglycerides (TG), high-density lipoprotein (HDL), and low-density lipoprotein (LDL) were determined according to the previously described methods [90,91,92,93], respectively, using commercial diagnostic kits supplied by Vitro Scient. Co., Cairo, Egypt.

### 4.9. Evaluation of Serum Tumor Markers

Alpha-fetoprotein (AFP) was measured in serum using ELISA Kit specific for rat AFP from CUSABIO Co. (Wuhan, China; Cat. No. CSB-E08281r).

### 4.10. Evaluation of Hepatic Oxidative Stress Biomarkers

Malondialdehyde (MDA) was assessed in the hepatic tissue using the technique of Draper and Hadley [94]. Estimation of tissue superoxide dismutase (SOD) depends on the ability of the enzyme to inhibit phenazine [95]. However, total glutathione (tGSH), oxidized glutathione (GSSG), reduced (GSH), and contents were estimated [96]. Following the manufacturer’s guidelines, all the previous parameters were measured spectrophotometrically using kits from Bio-Diagnostic Co., Cairo, Egypt.

### 4.11. Hepatic Apoptotic Markers Assessment

Apoptosis regulator BAX (BAX) was quantitatively assessed in hepatic homogenate using a rat BAX ELISA Kit from CUSABIO Co. (Cat. No. CSB-EL002573RA). Additionally, for the quantitative estimation of rat B-cell CLL/lymphoma 2 (BCL2), Rat BCL2 ELISA Kit from CUSABIO Co. (Cat. No. CSB-E08854r) was used. All procedures were carried out in compliance with the guidelines provided by the manufacturer.

### 4.12. Western Blot Analysis

Total proteins were extracted from tissue homogenates using ReadyPrep™ kit (Bio-Rad, Hercules, CA, USA) and quantified by Bradford assay. Equal amounts of protein (20 µg) were denatured in Laemmli buffer, separated by SDS-PAGE (Bio-Rad TGX Stain-Free gels), and transferred to PVDF membranes using the Trans-Blot Turbo system. Membranes were blocked with 3% BSA/TBST, incubated overnight at 4 °C with primary antibodies (Santa Cruz, Cell Signaling, Proteintech, Novusbio, Centennial, CO, USA), and then with HRP-conjugated secondary antibody. Protein bands were visualized using Clarity™ ECL substrate (Bio-Rad) and quantified with ChemiDoc MP Imaging System, normalized to β-actin [97].

### 4.13. Histopathological Assessment

The rats’ thigh muscle and hepatic tissue specimens were carefully dissected and submerged for 24 h in a 10% neutral buffered formalin solution. Thereafter, the samples are subjected to histological processing via a conventional paraffin embedding technique. The specimens are subsequently sectioned into 4 µm slices and stained with Mayer’s hematoxylin and eosin (H&E) [98].

Necrotic tumor score was measured on a scale of 0–4, where 0 meant there was no necrosis, 1 meant there was mild necrosis (0–20%), 2 meant there was severe necrosis (21–50%), 3 meant substantial necrosis (51–80%), and 4 widespread necrosis (the most severe, 81–100%) and significant necrosis (51–80%) [99]. In addition to necrosis, other histological indicators such as inflammatory infiltration, apoptosis, angiogenesis, and cytological characteristics were assessed. These alterations were categorized using negative (−), mild (+), moderate (++), and severe (+++) intensity levels [99,100].

Semiquantitative liver lesion scoring for vacuolar degeneration and lobular inflammation was scored as follows: 0 = normal; 1 = 25%; 2 = 26–50%; 3 = 51–75%; 4 = 76–100%. Ten randomly chosen photomicrographs per rat were taken per 100x field [101].

### 4.14. Immunohistochemical Protein Assay

Immunohistochemical staining for IL-1β was conducted using the streptavidin-biotin peroxidase technique. Hepatic tissue slides were deparaffinized with xylene for 24 h and subsequently rehydrated using a descending gradient of ethyl alcohol. A citric acid buffer (10 mM/L, pH 6) was applied to the slides to retrieve the antigen after microwave exposure. They were then allowed to cool and rinsed in PBS. By first incubating the slides with 3% H_2_O_2_ for 15 min and then using a mouse monoclonal antibody for 30 min at room temperature, the endogenous peroxidase activity was suppressed. The tissue sections were then exposed to diaminobenzidine (DAB) chromogen for ten minutes before being washed with PBS. In the end, the sections were cover-slipped, soaked in xylene, washed with water, dehydrated using graded ethanol concentrations, counterstained with hematoxylin for two minutes, and examined under a light microscope [102]. The Image J software (Version 2.16.0, National Institutes of Health, Bethesda, MD, USA) was utilized for quantifying immunostaining intensities. The area % was estimated as documented by Vis et al. [103] in 10 chosen micrographs from various regions of eight rats per group.

### 4.15. Electron Microscope Assessment

Fresh tissue samples, about 1 mm^3^ in size, were taken from the skeletal muscles and immediately fixed in 4F 1G at pH 7.2 and stored at 4 °C, after a cardiovascular perfusion of 4F 1G (2% formaldehyde, 1.25% gluteraldehyde in 0.1 M sodium cacodylate, PH 7.2). After that, for two hours, the samples were rinsed in 0.1 M phosphate buffer at a temperature of 4 °C every 15 min. A 1% solution of phosphate-buffered osmium tetroxide (2% osmic acid 5 mL and phosphate buffer 5 mL) was then used to post-fixate the samples for two hours at room temperature. Following that, the samples were quickly placed in increasing ethyl alcohol series grades (30, 50, 70, 90, and 100% for two changes) for 30 min each to dehydrate them. After that, it was put in a 1:1 propylene and epoxy resin combination and left overnight in propylene oxide. Epoxy resin was then used to embed them. The tissue blocks and embedding mixture were polymerized in an oven for five days in the following order: for twenty-four hours at 35 °C, twenty-four hours at 45 °C, and three days at 60 °C. To choose which parts would be investigated under an electron microscope, semithin slices (1 µm) were first cut, dyed with toluidine blue, and inspected under a light microscope. After cutting the ultrathin slices (60–100 nm) with a glass knife on an LBK ultramicrotome, they were stained with lead citrate 48 and uranyl acetate. The transmission electron microscope JEM-1400 PLUS, which is located in the electron microscopy unit of Alexandria University’s Faculty of Science, was used to analyze the sections [104].

### 4.16. Statistical Analysis

One-way analysis of variance (ANOVA) and post hoc analysis (Tukey’s test) at *p* < 0.05 are used to examine the results, which are shown as mean ± S.E.M. Data were analyzed via using GraphPad InStat version 10.3.1 (GraphPad Software, San Diego, CA, USA).

## 5. Conclusions

Nano-Gefitinib decreased Ehrlich-induced hepatic injury, oxidative stress, and apoptosis in mice. Current study shows improved hepatic histology and lower serum hepatic enzymes, restored redox homeostasis, and corrected lipid profile.

M2-like polarized tumor-associated macrophages (TAMs) are crucial for stimulating the angiogenesis, invasion, proliferation, and metastasis of cancer cells. Consequently, the detection of M2-like TAMs as tumors grow is a promising strategy for cancer therapy. As far as we are aware, this work is a groundbreaking attempt to assess the anticancer effects of Gefitinib nanoparticles in the ESC mice model. The results recorded that Gefitinib nanoparticles target macrophage polarization, angiogenesis, and apoptosis, which are vital processes involved in the development of cancer. By modifying EGFR-mediated anti-angiogenic, antiproliferative, and apoptotic properties, besides macrophage reprogramming, Gefitinib nanoparticles promoted tumor cell death. Consequently, Gefitinib nanoparticles are a promising therapeutic agent for the treatment of solid tumors like ESC.

## Figures and Tables

**Table 1:** Polymeric Nano-Gefitinib characteristics.

Parameter	Mean	Range
Particle size (nm)	113.66 ± 2.23	112 to 116.2
Zeta potential (mV)	−53.68 ± 1.36	−54.91 to −52.22
PDI	0.31 ± 0.02	0.294 to 0.333
Drug content (mg/mL)	3.13 ± 0.24	3.31 to 2.87
%EE	94.03 ± 7.05	86.02 to 99.31

**Table 2:** Histopathological scores of different experimental groups.

Parameters	Control	Nano-Gefitinib	Ehrlich	Combination
Necrosis score	−	−	0	4
Cytological features	−	−	+++	+
Apoptosis	−	−	+	+++
Angiogenesis	−	−	+++	+
Inflammatory infiltrates	−	−	+++	+

Grades of necrosis were as follows: 0 denoted no necrosis, 1: mild necrosis (0–20%), 2—moderate necrosis (21–50%), 3—pronounced necrosis (51–80%), and 4—the most severe degree (81–100%). Negative (−), mild (+), moderate (++), and severe (+++).

## Data Availability

The original contributions presented in this study are included in the article. Further inquiries can be directed to the corresponding author.

## Abbreviations

The following abbreviations are used in this manuscript:

EGFR-TKIs	Epidermal growth factor receptor-tyrosine kinase inhibitors
TG	Triglyceride
LDL	Low density lipoprotein
HDL	High density lipoprotein
TGF	Transforming growth factor
NF-κB	Nuclear factor kappa B
VEGF	Vascular endothelial growth factor
MMP	Matrix metalloproteinase
